# Infrared Fluorescent Protein 1.4 Genetic Labeling Tracks Engrafted Cardiac Progenitor Cells in Mouse Ischemic Hearts

**DOI:** 10.1371/journal.pone.0107841

**Published:** 2014-10-30

**Authors:** Lijuan Chen, M. Ian Phillips, Hui-Lai Miao, Rong Zeng, Gangjian Qin, Il-man Kim, Neal L. Weintraub, Yaoliang Tang

**Affiliations:** 1 Department of Cardiology, Zhongda Hospital, Medical School of Southeast University, Nanjing, China; 2 Keck Graduate Institute, Claremont, California, United States of America; 3 Affiliated Hospital of Guangdong Medical College, Zhanjiang, China; 4 Feinberg Cardiovascular Research Institute, Northwestern University Feinberg School of Medicine, Chicago, Illinois, United States of America; 5 Department of Medicine, University of Cincinnati, Cincinnati, Ohio, United States of America; 6 Vascular Biology Center, Department of Medicine, Medical College of Georgia, Georgia Regents University, Augusta, Georgia, United States of America; Second University of Naples, Italy

## Abstract

Stem cell therapy has a potential for regenerating damaged myocardium. However, a key obstacle to cell therapy’s success is the loss of engrafted cells due to apoptosis or necrosis in the ischemic myocardium. While many strategies have been developed to improve engrafted cell survival, tools to evaluate cell efficacy within the body are limited. Traditional genetic labeling tools, such as GFP-like fluorescent proteins (eGFP, DsRed, mCherry), have limited penetration depths in vivo due to tissue scattering and absorption. To circumvent these limitations, a near-infrared fluorescent mutant of the DrBphP bacteriophytochrome from Deinococcus radiodurans, IFP1.4, was developed for in vivo imaging, but it has yet to be used for in vivo stem/progenitor cell tracking. In this study, we incorporated IFP1.4 into mouse cardiac progenitor cells (CPCs) by a lentiviral vector. Live IFP1.4-labeled CPCs were imaged by their near-infrared fluorescence (NIRF) using an Odyssey scanner following overnight incubation with biliverdin. A significant linear correlation was observed between the amount of cells and NIRF signal intensity in in vitro studies. Lentiviral mediated IFP1.4 gene labeling is stable, and does not impact the apoptosis and cardiac differentiation of CPC. To assess efficacy of our model for engrafted cells in vivo, IFP1.4-labeled CPCs were intramyocardially injected into infarcted hearts. NIRF signals were collected at 1-day, 7-days, and 14-days post-injection using the Kodak in vivo multispectral imaging system. Strong NIRF signals from engrafted cells were imaged 1 day after injection. At 1 week after injection, 70% of the NIRF signal was lost when compared to the intensity of the day 1 signal. The data collected 2 weeks following transplantation showed an 88% decrease when compared to day 1. Our studies have shown that IFP1.4 gene labeling can be used to track the viability of transplanted cells in vivo.

## Introduction

Recent studies show that stem/progenitor cells may regenerate cardiac tissue directly by inducing neovasculogenesis and cardiogenesis [1]. Resident cardiac progenitor cells are particularly suitable for resurrecting dead myocardium because they are endogenous components of the adult heart and appear to be responsible for the physiologic and pathologic turnover of cardiac myocytes and other cardiac cells [2]–[6].

Poor cell survival and retention are two of the primary barriers to the effectiveness of cell therapy [7], [8]. Many techniques have been used to enhance the survival of transplanted stem/progenitor cells to maximize its regenerative potential [5], [7]; yet, a method for monitoring engrafted cell survival and biodistribution in real time remains a challenge for determining optimum dosing strategies [9]. Application of conventional GFP-like fluorescent proteins, including eGFP, DsRed, and mCherry, for imaging of mammals is limited by the penetration depths of visible light in the body [10]. However, proteins with excitation and emission maxima within a near-infrared window from ∼650 nm to 900 nm can be used for in vivo imaging as they have lower absorbance and scattering in tissues [11], [12]. Tsien and his colleagues [13] developed near-infrared fluorescent protein (IFP) from the DrBphP bacterial phytochrome of Deinococcus *radiodurans*. They discovered a mutant form named IFP1.4, to be powerful for in vivo imaging of adenovirus infected mammalian liver. Yu D and his colleagues [13] recently used IFP labeling to image brain tumor in mice, and show promise in the application of IFPs for protein labelling and in vivo imaging.

As there is no report about using IFP1.4 for stem cell tracking in vivo, in this study, we tracked sca-1+ cardiac progenitor cells (CPC) in vivo using a lentiviral-IFP1.4 vector, which allowed us to track labeled cells permanently until the cells died [14]. We found that the IFP1.4-labeled CPCs can be readily detected in the infarcted hearts noninvasively in vivo after biliverdin injection. The function of biliverdin is the chromophore for IFP1.4, which can spontaneously incorporate biliverdin [15]; thus, lentiviral-IFP1.4 cell labeling technology opens a novel approach for monitoring stem cell homing and survival in living animals. These findings broaden the application of IFP1.4 system for stem cell studies.

## Materials and Methods

### Ethics Statement

All animal care and use was approved by the University of Cincinnati and Georgia Regents University Institutional Animal Care and Use Committee (IACUC).

### Cardiac progenitor cell (CPC) culture

Mouse Sca-1+ CPC were isolated as previously described [2], [3], [5] using a 2-step protocol approved by the Institutional Animal Care and Use Committee of the University of Cincinnati and Georgia Regents University. Minced murine hearts were plated on laminin-coated dishes for 2 weeks. The round, phase-bright migrating cells were harvested and filtered with 40 µm cell strainers to avoid heart tissue contamination. Sca-1+ cells were isolated through the use of a hematopoietic Lin-depletion cocktail (StemCell Technologies, Vancouver, BC, Canada) followed by magnetic-activated cell sorting with sca-1 magnetic beads (Miltenyi Biotec Inc, Auburn, Calif) as instructed by the protocols of the manufacturers. The selected CPCs were cultured in complete CPC media containing Dulbecco’s modified Eagle’s medium (DMEM)/F12, 10% fetal calf serum, 2 mM L-glutamine final concentration, 55 µM ß-mercaptoethanol, and 1x MEM nonessential amino acids (NEAA) (Invitrogen Corporation, Carlsbad, CA).

### Flow cytometry

Flow cytometry analyses of cultured CPC were performed with a BD LSRII flow cytometer. Briefly, CPC cells were blocked with 5% rat serum and stained with a panel of conjugated antibodies, including anti-Sca-1-PE, anti-CD31-biotin,, anti-c-kit-biotin, anti-CD45-PE-cy7, anti-Flk1-PerCP-cy5.5 and anti-CD105-V450 (BD Biosciences, San Jose, CA), or isotype-matched control antibodies. Anti-c-kit-biotin, and anti-CD31-biotin antibodies were resolved via secondary staining with streptavidin-PerCP-Cy5.5 or streptavidin-FITC, respectively.

### Lenti-IFP1.4 vector construction, and lentiviral infection of CPC

The pSin-EF2-IFP1.4-Puro plasmid was created by amplifying a 1 kb fragment of IFP1.4 cDNA from pcDNA3-IFP1.4 (Gift from Roger Y. Tsien) into EcoR1 and Spe1 in pSin-EF2-Lin28-Pur (Addgene 16580, 5′ PCR primer: CTAG GAATTC AAGCTTGCCACCATGGCTCG. 3′ PCR primer: CTAG ACTAGT TCATTTATACAGCTCGTCCA). Viral vectors encoding IFP1.4 were produced by transfection of 293FT cells with the lentiviral backbone plasmid pSin-EF2-IFP1.4-Puro, an envelope plasmid (pMD2.G), and a packaging plasmid (psPAX2) with Fugene HD (Roche Diagnostics GmbH, Mannheim, Germany). Virus-containing medium was collected 48 h after transfection on 2 consecutive days, passed through a 0.45 µm filter to remove cell debris, and concentrated by ultracentrifugation. Lentiviral vectors expressing IFP1.4 with 8 µg/mL polybrene (Sigma-Aldrich, St. Louis, MO) were applied to mouse CPC cells; At 72 h after infection, the medium was replaced. Transduced cells were selected with 5, 7.5 and 10 µg/mL puromycin beginning on the third day after infection and continuing until 1 week after all mock-transfected cells had died or throughout the generation of stable, clonal, transduced cell lines. After puromycin selection, IFP1.4 positive CPC were examined after overnight incubation with 25 µM biliverdin (Frontier Scientific) in DMEM. The plates were scanned using the Odyssey Infrared Imager at 700 nm channel (Li-Cor).

### TUNEL assay

To determine whether IFP 1.4 infection impacts on cell apoptosis, cells were plated on 8-well chamber slides (Millipore, Billerica, MA). The terminal deoxynucleotidyltransferase-mediated dUTP-biotin nick-end-labeling (TUNEL) staining for apoptotic nuclei was performed using DEAD End TUNEL kit (Promega, Madison, WI) according to the manufacturer’s instructions with minor modifications. Briefly, cells were fixed in 4% paraformaldehyde for 25 min and then treated with permeabilization solution (0.2% Triton X-100 solution in PBS) for 5 min at room temperature. Labeling reactions were performed with 100 µl of reaction buffer for 60 min at 37°C in a humidified chamber, followed by steptavidin Alexa Fluor 555 conjugate (1∶400, Life Technologies, Carlsbad, CA) staining. Slides were mounted using VECTASHIELD HardSet Mounting Medium with DAPI (Vector Laboratories, Burlingame, CA). Apoptosis was evaluated as the average number of TUNEL-positive cells per DAPI labeled cells at high-power magnification.

### In Vitro Differentiation and Quantitative Reverse Transcription Polymerase Chain Reaction (QRT-PCR)

Trichostatin (TSA) has been reported to promote cardiomyocyte differentiation of mesenchymal stem cells [16]–[18]. To determine whether IFP1.4 gene modification changes cardiomyocyte differentiation of CPC, the cultured CPC ^Control^ and CPC ^IFP1.4^ were cultured in gelatin coated 6 well plates, and received treatments as following: 1) Control DMSO; 2) TSA (50 nmol/L) for 24 hrs. Total RNA from CPC ^Control^ and CPC ^IFP1.4^ was extracted by RNAzol RT (Molecular Research Center, Inc. Cincinnati, OH) following the manufacturer’s instructions. 1st strand cDNA was synthesized using the PrimeScript 1^st^ strand cDNA synthesis kit (TakaRa Biotechnology). The cDNA synthesized was used to perform quantitative PCR on an Mx3000P Real-Time PCR System (Agilent Technologies, Santa Clara, CA) using the SensiMix SYBR kit (Bioline, Tauton, MA). Amplification was performed at 95°C for 10 min, followed by 40 cycles of 95°C for 15 s and 60°C for 1 min. Primers used for each specific product are as follows: cardiac TnI (Tnni3): Forward: CACCTCAAGCAGGTGAAGAA, Reverse: GCCACTCAGTGCATCGATATT, GAPDH: Forward: TCAACAGCAACTCCCACTCTTCCA, Reverse: ACCCTGTTGCTGTAGCCGTATTCA.

### Myocardial infarction, and in vivo NIRF imaging of intramyocardial injected CPC

Male C57/BL6 mice were anesthetized with ketamine/xylazine (100 mg/kg/10 mg/kg, i.p.) and mechanically ventilated. Myocardial infarction was induced via ligation of the left anterior descending coronary artery 2 mm from the tip of the normally positioned left atrium as we described previously [2], [5], [7], [19]. A 25 µl solution containing 5×10^5^ CPC ^IFP1.4^ in DMEM was injected at one site intramyocardially with 31 gauge needle immediately after induction of MI. Animals were handled according to approved protocols and animal welfare regulations of the Institutional Animal Care and Use Committee of the University of Cincinnati and the Medical College of Georgia. 1 day, 7 days and 14 days after cell transplantation, MI mice were intravenously injected with 250 nmole biliverdin via tail vein, and whole animal near infrared fluorescence imaging was performed under general anesthesia (isoflurane) inhalation using a Carestream MultiSpectral FX (2D) system in epifluorescence mode equipped with 620/20 nm (center wavelength/full width at half maximum) and 720/20 nm filters for excitation and emission. The signal intensity is represented by radiance and encoded by pseudocolors on the IFP1.4 image. The signal intensity is represented by radiance and encoded by pseudocolors on the IFP1.4 image.

## Results

### Phenotypic Characterization of CPC Cells

**Figure 1:**
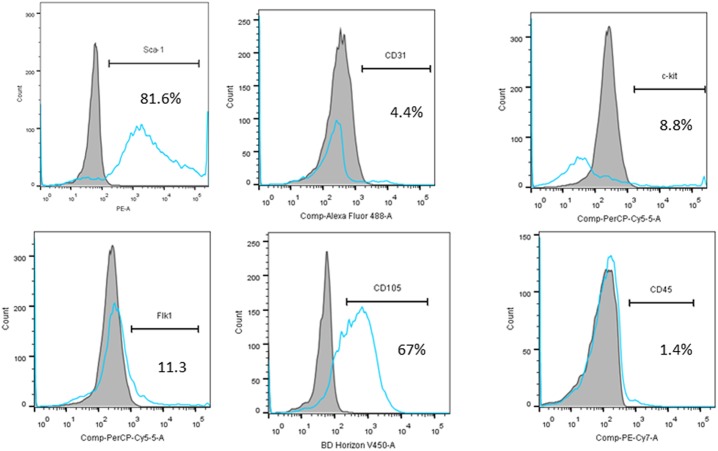
Flow cytometric analyses of CPC cells for expression of the cell surface markers Sca-1, CD31, c-kit, Flk-1, CD105 and CD45.

CPC cells were obtained with a 2-step procedure. After sorting, surface marker expression was profiled by flow cytometry. As demonstrated in **Figure 1**, more than 80% of the sorted cells were positive for sca-1 expression, and more than 65% of the sorted cells were positive for CD105, smaller populations of the CPC cells expressed Flk-1 (11.3%), c-kit (8.8%), or CD31 (4.4%), and <1.5% of cells expressed the hematopoietic lineage marker CD45.

### In vitro quantification of IFP1.4 labeled CPCs by near infrared signal

**Figure 2:**
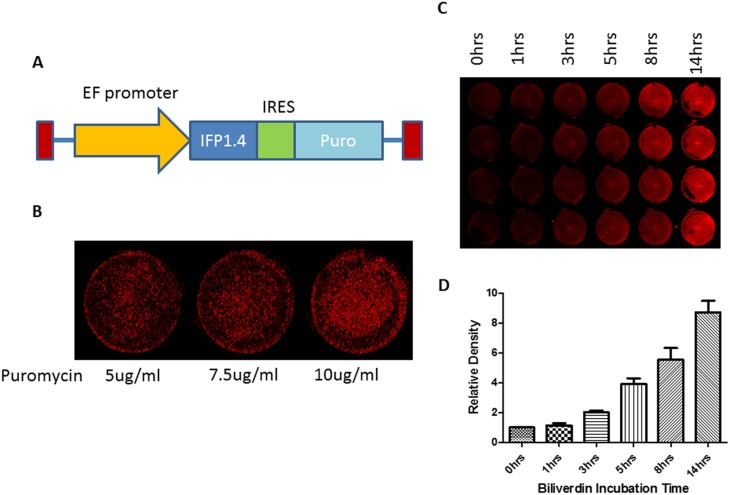
Intensity of near infrared signal in CPC after incubation with bilirubin in vitro. (**A**) Schematic of the lenti- IFP1.4 construction; (**B**) lentiviral vector was used to genetically label IFP1.4 in mouse CPC, After CPC infected with lentivirus-IFP1.4 for 3 days, we used Puromycin at dosage of 5, 7.5, 10 µg/ml to select the CPC; (**C–D**) comparing near infrared signals from CPC ^IFP1.4^ incubated with bilirubin (25 µm) from 0∼14 hrs. CPC ^IFP1.4^ were incubated with 25 uM biliverdin for 0 hrs, 1 hrs, 3 hrs, 5 hrs, 8 hrs and 14 hrs, n = 4.

**Figure 3:**
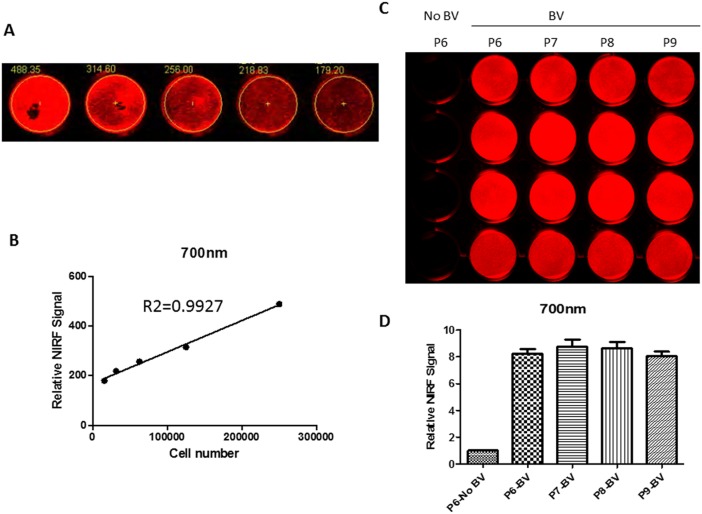
Assessment of signal consistence of IFP1.4 labeled CPC. (**A**) Correlation between CPC numbers and near infrared signals, IFP1.4-labeled CPC at 2.5×10^5^, 1.25×10^5^, 6.25×10^4^, 3.125×10^4^, and 1.57×10^4^ were seeded into 24-well plate, and incubated with 25 µM biliverdin overnight, and then subjected to scanning on the Odyssey Infrared imager; (**B**) Assessment of near infrared signal intensity showed a robust linear correlation (R^2^ = 0.9927) between the cell number and near infrared signal; (**C–D**) Comparison of near infrared signal from CPC ^IFP1.4^ at P6, P7, P8 and P9 with/without Biliverdin treatment.

We have constructed a lentiviral vector containing IFP1.4 gene to stably express IFP1.4 protein in mouse CPC (**Fig. 2A**), in order to purify the IFP1.4-expressing CPC, we subjected them to puromycin selection at the concentration of 5, 7.5 and 10 ug/ml, as shown in **Fig. 2B**, selection by puromycin at the highest dose (10 µg/ml) gave high proportion of IFP1.4-positive cells with high level of fluorescent signals. Next, we incubated IFP1.4-positive cells (selection with 10 µg/ml puromycin) with biliverdin for extended period of time, ranging from 0–14 hrs; obvious IFP1.4 signals were observed after a 5-hour incubation when 25 µM biliverdin was applied. The strongest signal appears after cell exposure at 14 hrs (**Fig. 2C–D**), suggesting time dependent IFP1.4 signal. Finally, we investigate whether IFP1.4 labeling can be used for cell quantification accurately. First, we evaluated the correlation between near infrared signal and the amount of seeding cells. We observed a significant linear correlation existed between the amount of cells (x) and near infrared signal values (y). The square of the correlation coefficient (R2) was 0.9927 (**Fig. 3A–B**), which suggests that IFP1.4 molecular signal enables accurate cell quantification in vitro – an important feature for assessing cell proliferation. Persistence of IFP1.4 signal from labeled cells is important for us to determine whether this technology can be used for assaying in vivo stem cell survival, we evaluated the persistence of the IFP1.4 fluorescent signal of CPC for four passages (P6-P9) with time interval at 1 week intervals. As demonstrated in **Fig. 3C–D**, lentiviral mediated IFP1.4 gene labeling shows persistent signal at least three weeks without decay, suggesting that IFP1.4 molecular labeling enables real-time assessment of cell survival in vivo.

### Genetic labeling with IFP1.4 does not change CPC apoptosis

**Figure 4:**
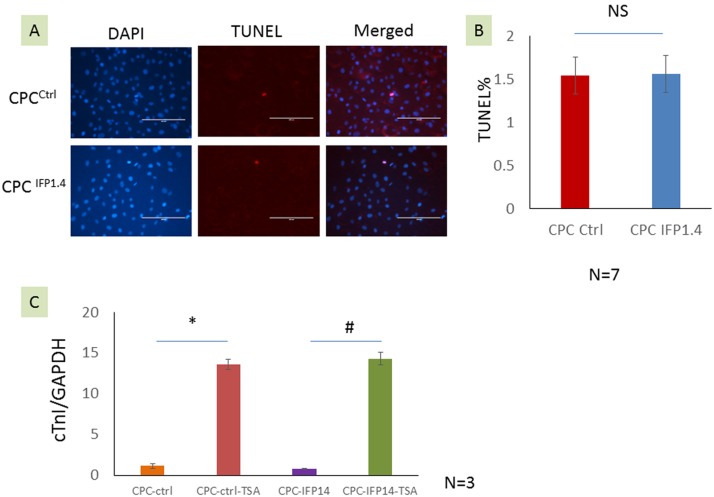
Analysis of CPC apoptosis and cardiac differentiation capacity after IFP1.4 gene modification. (**A–B**) Comparison of cell apoptosis between un-infected CPC and IFP1.4 infected CPC by TUNEL staining; (**C**) Real time RT-PCR comparison of cTnI expression between un-infected CPC and IFP1.4 infected CPC with/without TSA treatment.

To determine whether genetic labeling with IFP 1.4 impacts on cell survival, we compare the apoptotic cells between un-infected and IFP1.4 infected CPC by TUNEL assay, there is no difference in cell apoptosis between two groups of cells (**Fig. 4A–B** **, P>0.05, n = 7**).

### Genetic labeling with IFP1.4 does not change cardiac differentiation capacity of CPC

To determine whether IFP1.4 infection impacts on cell differentiation, we treated CPC with TSA, a HDAC inhibitor, to induce cardiac differentiation of CPC. We compared cardiac troponin I expression between un-infected CPC and IFP1.4 infected CPC with/without TSA treatment, we found that IFP1.4 modified CPC show similar response to TSA in comparison with un-infected CPC (**Fig. 4C**).

### Tracking survival of engrafted CPC using near infrared fluorescent (NIRF) imaging in ischemic heart In vivo

**Figure 5:**
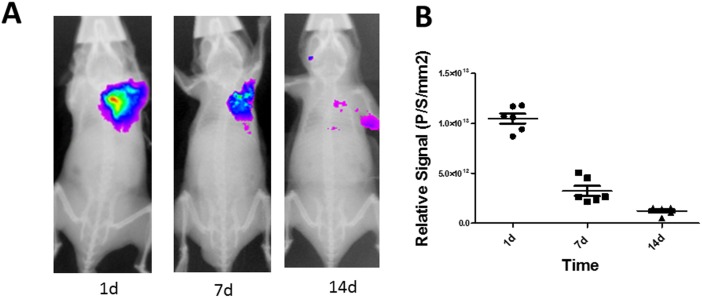
Assessment of CPC survival in ischemic myocardium using in vivo near infrared fluorescent (NIRF) imaging. (**A–B**) Representative near-infrared fluorescence imaging in mice obtained at 1d, 1w and 2w post cell transplantation (n = 6), the fluorescence image was overlaid on an X-ray using a KODAK In-Vivo Multispectral FX Image 2D Station.

To determine whether lenti-IFP1.4 cell labeling can be used for in vivo tracking of cell survival in ischemic hearts following cell transplantation, CPCs were genetically modified by lentiviral vector with IFP1.4 (CPC ^IFP1.4^). Mice then underwent left anterior descending artery ligation and intramyocardial injection with CPC ^IFP1.4^. Noninvasive near infrared fluorescence and X-ray image overlays were conducted at day 1, day 7 and day 14 after cell transplantation to track the survival of transplanted cells in vivo. A strong near infrared signal was observed in mice treated with IFP1.4-labeled CPC at 1 day post cell transplantation; however, signal strength was significantly reduced by 70% 1 week post cell transplantation, and further reduced by 88% at 2 weeks post cell transplantation (**Fig. 5A–B** **, n = 6**).

## Discussion

Near-infrared based fluorescence imaging using IFP1.4 gene labeling is a novel technology with potential value for in vivo studies. In this study, we found that IFP1.4-labeled CPCs can be detected by their near infrared fluorescent signal using an Odyssey imaging scanner after incubation with biliverdin-containing media in vitro. The signal intensity is biliverdin-incubation time dependent; furthermore, a significant linear coefficiency between fluorescent signal and cell number in vitro exists, which suggests that IFP1.4 signal can reflect the number of seeding cells in vitro. We evaluate the persistence of the fluorescent signal in vitro, our data show there is no significant signal decay over 3 weeks, suggesting the signal persistence of the lenti-IFP1.4 labeled CPC, in addition, IFP1.4 labeling does not increase CPC apoptosis, and does not change cardiac differentiation capacity of CPC. Our studies demonstrate that IFP1.4-labeling CPC can be readily detected in mouse hearts at 1 day and 1 week after intramyocardial cell delivery. This suggests that IFP1.4 can be utilized for noninvasive, in vivo transplanted stem/progenitor cell tracking.

Hu S. et al [20] reported the survival course of engrafted mouse cardiac progenitor cells in ischemic myocardium by in vivo bioluminescence imaging as 8.0×10^4^±2.8×10^4^ at Day 2, 1.6×10^4^±9.5×10^3^ at Day 7, and 5.3×10^3^±3.3×10^2^ at Day 14. This suggests that 80% of engrafted CPCs are lost at 1 week after cell transplantation and that 93% are lost at 2 weeks when compared with the results from day 2. By using an IFP1.4 imaging system, we demonstrated that 70% of engrafted CPCs are lost at 1 week after cell transplantation, and 88% are lost at 2 weeks when compared to the data from day 1. Since our results using IFP1.4 approach are comparable to the results using firefly luciferase-based bioluminescence approach in evaluating engrafted cell survival in ischemic hearts, this demonstrates that the lenti-IFP1.4 labeling system is another option for monitoring the survival of engrafted progenitor cells in vivo.
